# Supplementary material for: Correlation and co-localization of QTL for stomatal density, canopy temperature, and productivity with and without drought stress in Setaria
Source: J Exp Bot. 2021 Apr 24;72(13):5024–37. doi: 10.1093/jxb/erab166 (PMC8219040; doi:10.1093/jxb/erab166)
Supplement: erab166_suppl_suppl_Supplementary_Figures [file erab166_suppl_suppl_supplementary_figures.pdf]

A10 B100

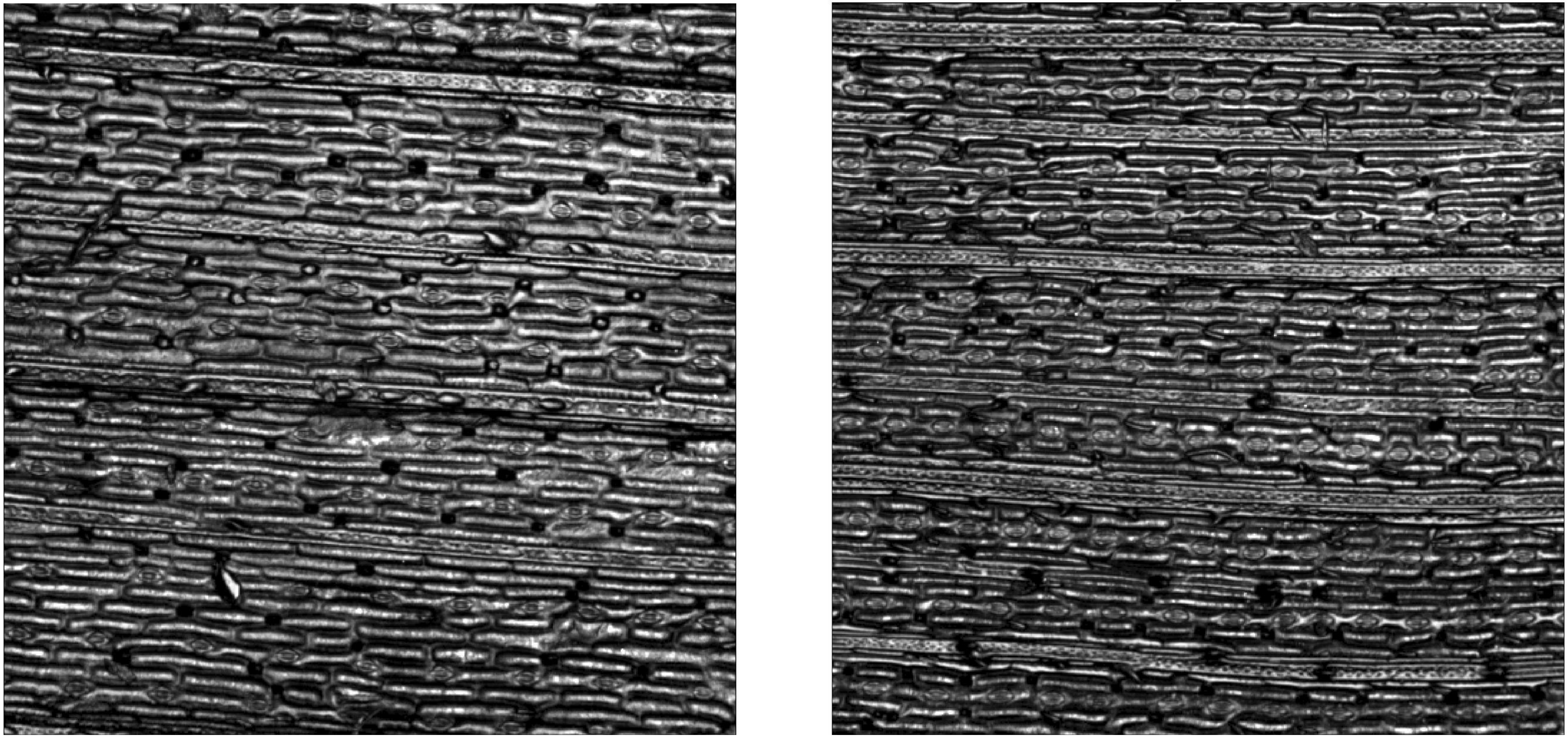

Supplemental Fig. S1. Representative images from optical tomography of abaxial leaf surfaces of *Setaria viridis* (A10) and *Setaria italica* (B100)

Supplemental data

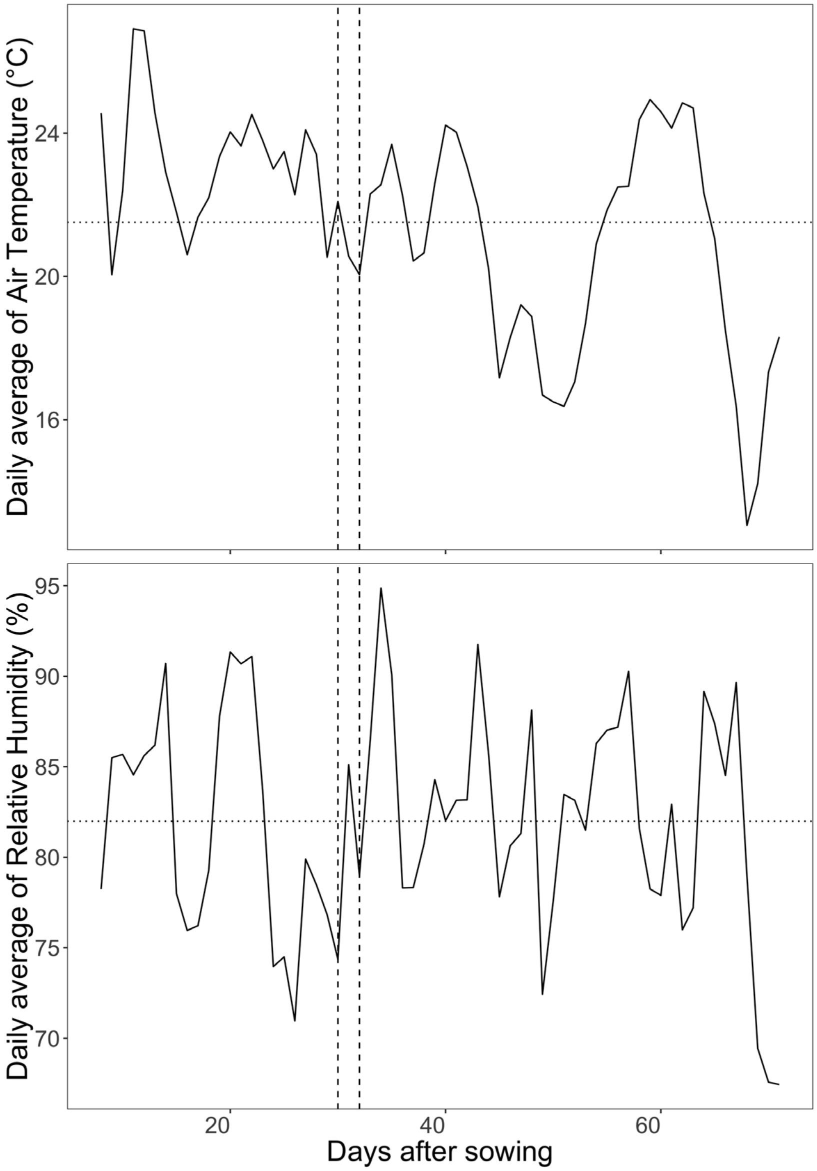

Supplemental Fig. S2. Daily average values of air temperature (a) and relative humidity (b) at SoyFACE experimental field site. The horizontal dotted line indicates the mean over the entire growing season. The vertical dashed lines indicate the days after sowing the canopy temperature measurements were collected in the field.

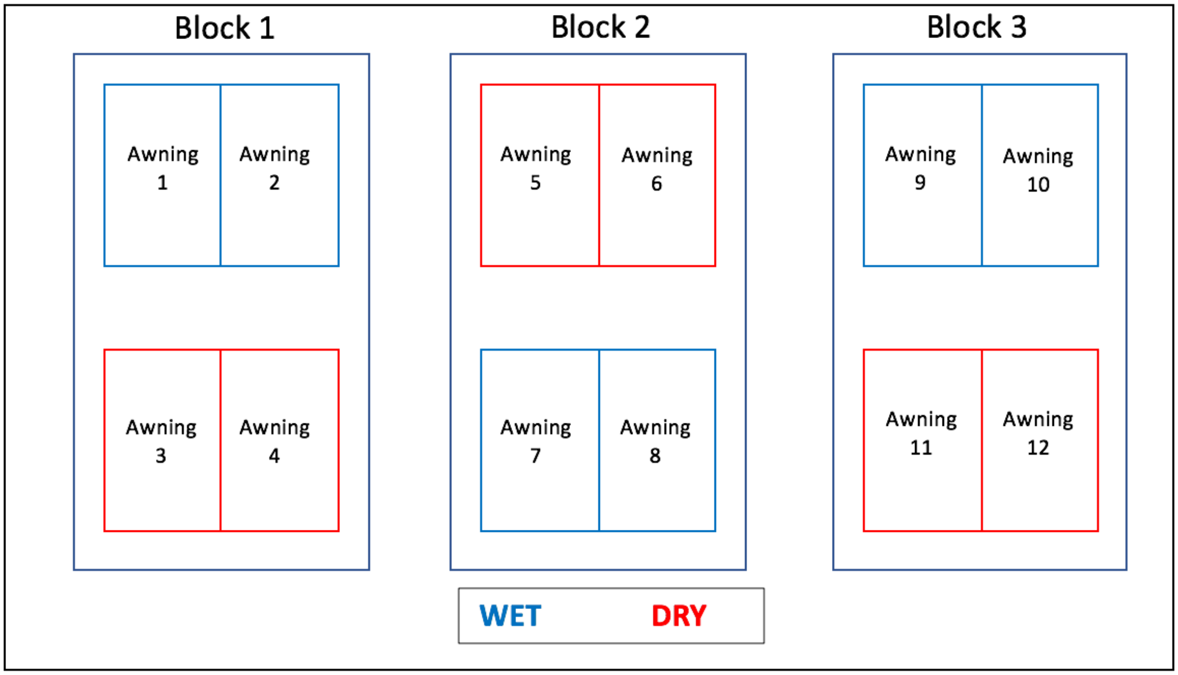

Supplemental Fig. S3. The layout of the field experiment with 3 blocks, 2 treatments, 12 awnings and 120 genotypes for each block in both treatments.

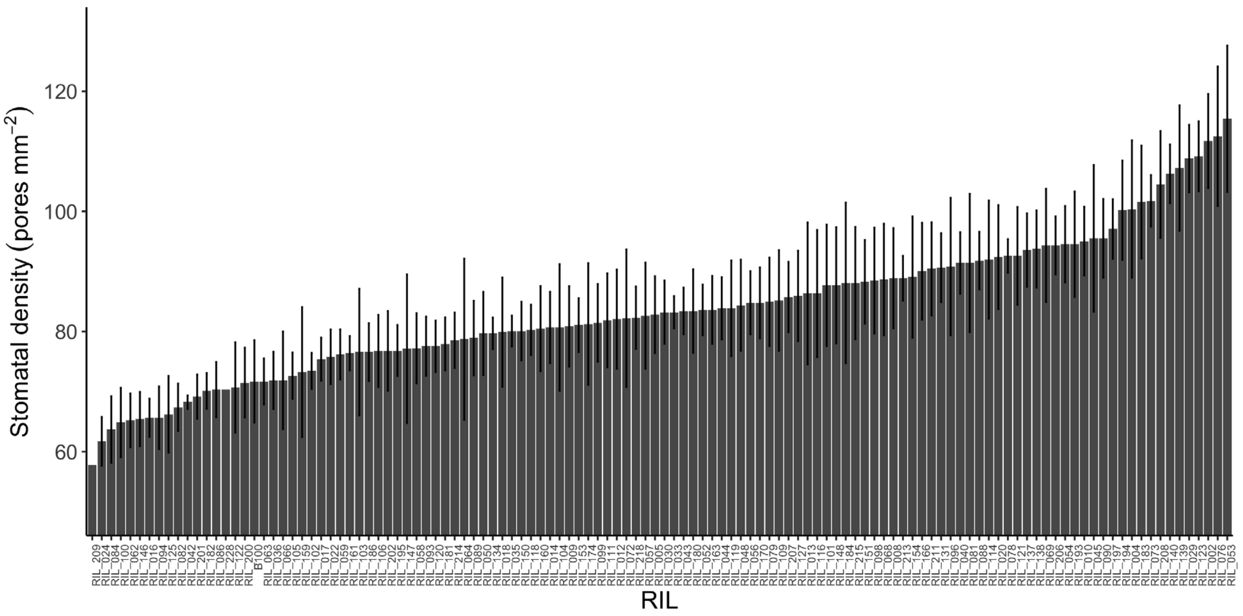

Supplemental Fig. S4. Stomatal density of 120 recombinant inbred lines derived from a cross of *S.italica* and *S.viridis, and B100 parental line.* Bars represent the genotype means (± standard error, n=4) derived from two fields of view from each of four replicate plants.

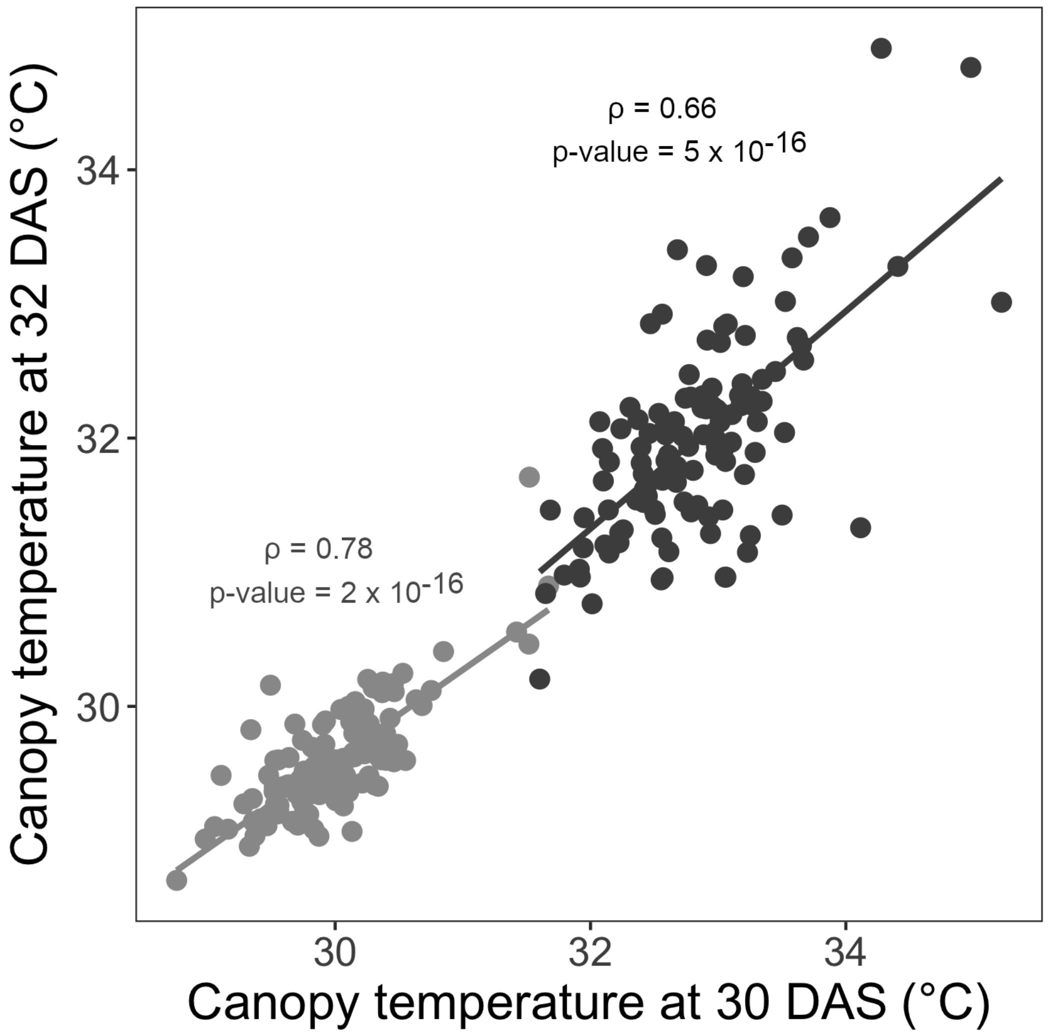

Supplemental Fig. S5. Scatterplot of midday canopy temperature for Setaria RILs and B100 on 30 DAS versus 32 DAS under wet (●) and dry treatments (●). Lines of best fit are shown along with the Pearson's correlation coefficient (ρ) and associated p-value.

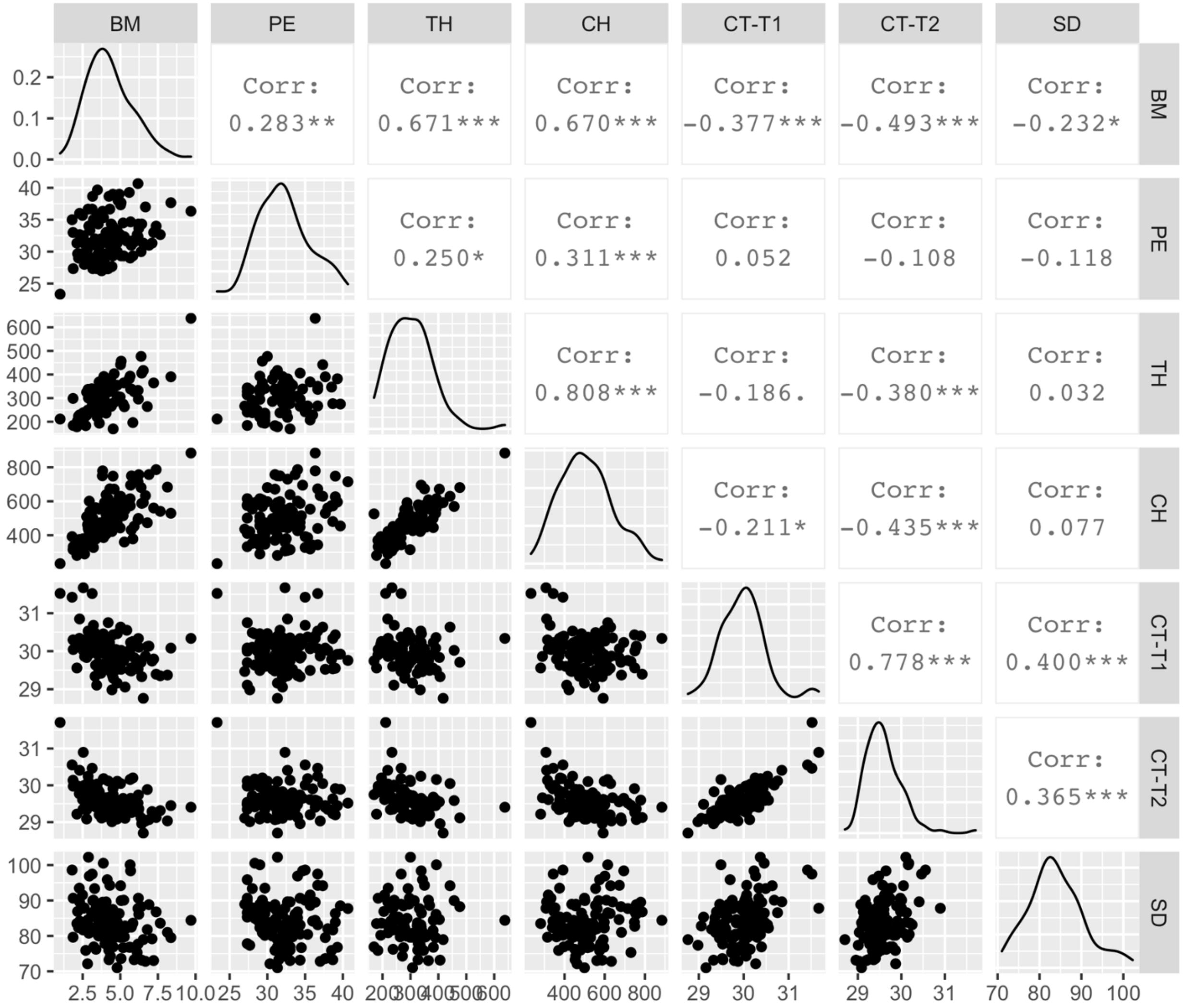

Supplemental Fig. S6. Phenotypic trait correlations of stomatal density (SD) versus canopy temperature at 30 DAS (CT-T1) and 32 DAS (CT-T2), total biomass (BM), panicle emergence (PE), culm height (CH) and tiller height (TH) under wet treatment condition in this study.

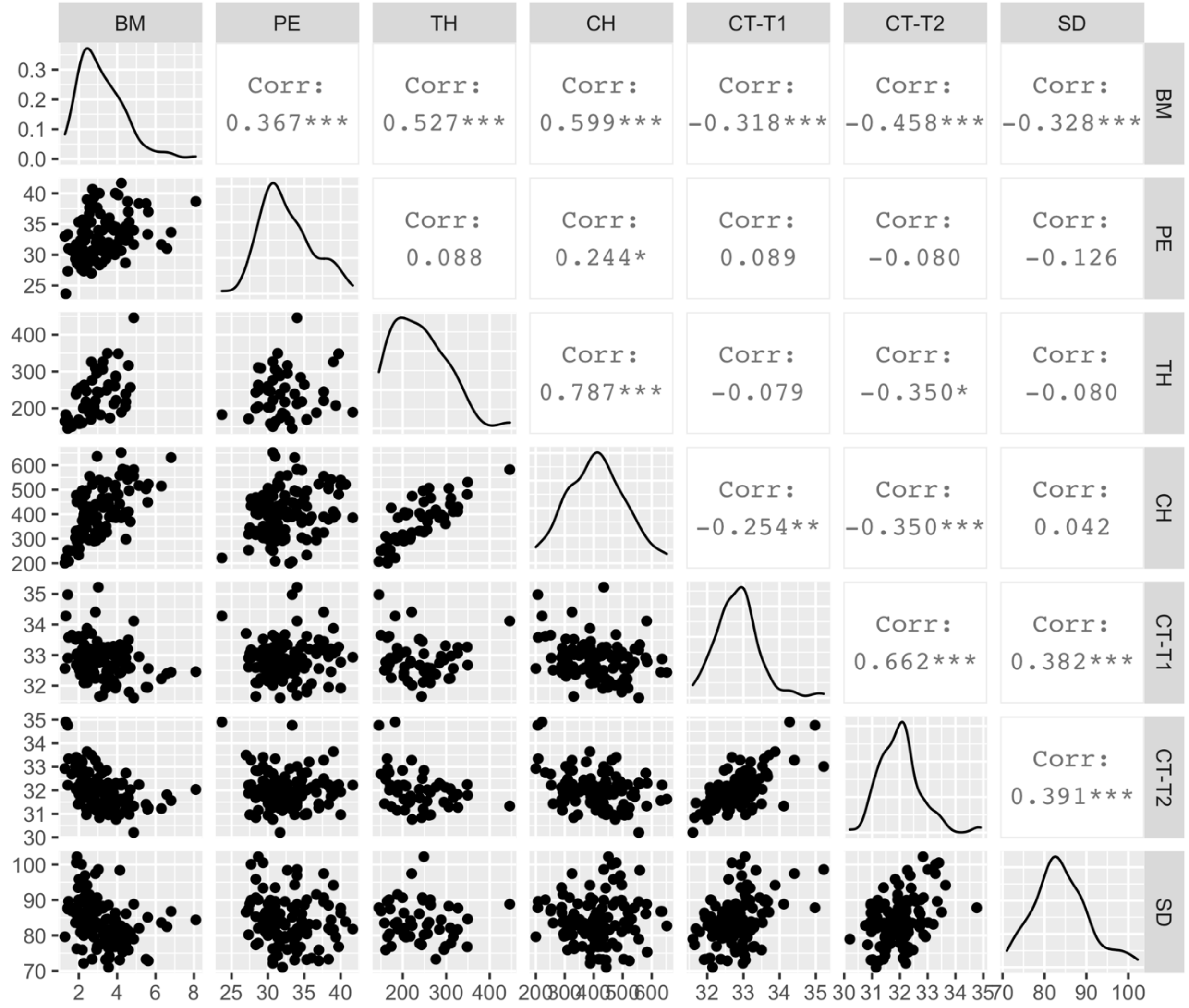

Supplemental Fig. S7. Phenotypic trait correlations of stomatal density (SD) versus canopy temperature at 30 DAS (CT-T1) and 32 DAS (CT-T2), total biomass (BM), panicle emergence (PE), culm height (CH) and tiller height (TH) under dry treatment condition in this study.
